# Meningitis Due to Apiotrichum mycotoxinivorans: A Rare Case Report

**DOI:** 10.7759/cureus.70573

**Published:** 2024-09-30

**Authors:** Ashok Kumar, Pankhuri Kumari, Manish Gaba

**Affiliations:** 1 Internal Medicine, Max Smart Super Specialty Hospital, New Delhi, IND; 2 Microbiology, Amrita Hospital Faridabad, Faridabad, IND

**Keywords:** first ever case, fungal meningitis, india, invasive fungal infections, ventriculoperitoneal (vp) shunt

## Abstract

A young adult male presented as a case of meningitis in the background of a ventriculoperitoneal shunt in situ, uncontrolled type 1 diabetes and a history of treated abdominal tuberculosis. The patient presented with complaints of high-grade fever, non-projectile vomiting, headache and drowsiness. He was eventually diagnosed as a case of fungal meningitis. The patient’s cerebrospinal fluid analysis (CSF) analysis revealed budding yeast cells and the culture revealed Apiotrichum mycotoxinivorans. This is a yeast-like fungus that is known to cause opportunistic infections in an immunocompromised host. This is a rare cause of fungal meningitis and very few cases have been reported worldwide. This is the first case of Apiotrichum mycotoxinivorans-associated meningitis reported from India.

## Introduction

Apiotrichum mycotoxinivorans (Trichosporon spp.) are basidiomycetous yeast-like anamorphic organisms widely distributed in nature and found predominantly in tropical and temperate areas [1]. Infections with invasive Trichosporon spp. are usually associated with central venous catheters, vesical catheters, and peritoneal catheter-related devices [2]. It is important to consider that Trichosporon spp. is widely distributed in nature and may be part of the normal biota of the skin, respiratory tract, gastrointestinal tract, and vagina. The ability to adhere to and form biofilms on implanted devices can account for the progress of invasive trichosporonosis [2], as it can promote the escape from antifungal drugs and host immune responses. This case represents an important opportunity to learn about a rare cause of fungal meningitis.

## Case presentation

A male patient in his 20s presented with a history of high-grade fever of 103 degrees Fahrenheit associated with recurrent non-projectile vomiting, headache and drowsiness for one day. He had a background history of type 1 diabetes, post-traumatic hydrocephalus, with a ventriculoperitoneal shunt (VP shunt) in situ and treated abdominal tuberculosis. He had type 1 diabetes for six years which has been uncontrolled with Hba1c-10 for one year. He has been on basal bolus regimen but his compliance has been inadequate over the last one year. He had a ventriculoperitoneal shunt inserted for a traumatic hydrocephalus one year back. He was diagnosed with abdominal tuberculosis 10 months back and had completed full course of anti-tubercular treatment which was taken for six months. The patient appeared lethargic on examination. He was hemodynamically stable with a normal cardiovascular, respiratory and per abdomen examination. His neurological examination revealed a Glasgow coma scale of E4V5M6. His higher mental function was normal. Meningeal signs were present with neck stiffness, Kernig’s and Brudzinski's signs were positive. He had a power of 5/5 in all four limbs. He denied any photophobia. A fundoscopy was done which was normal.

Investigation

**Figure 1:**
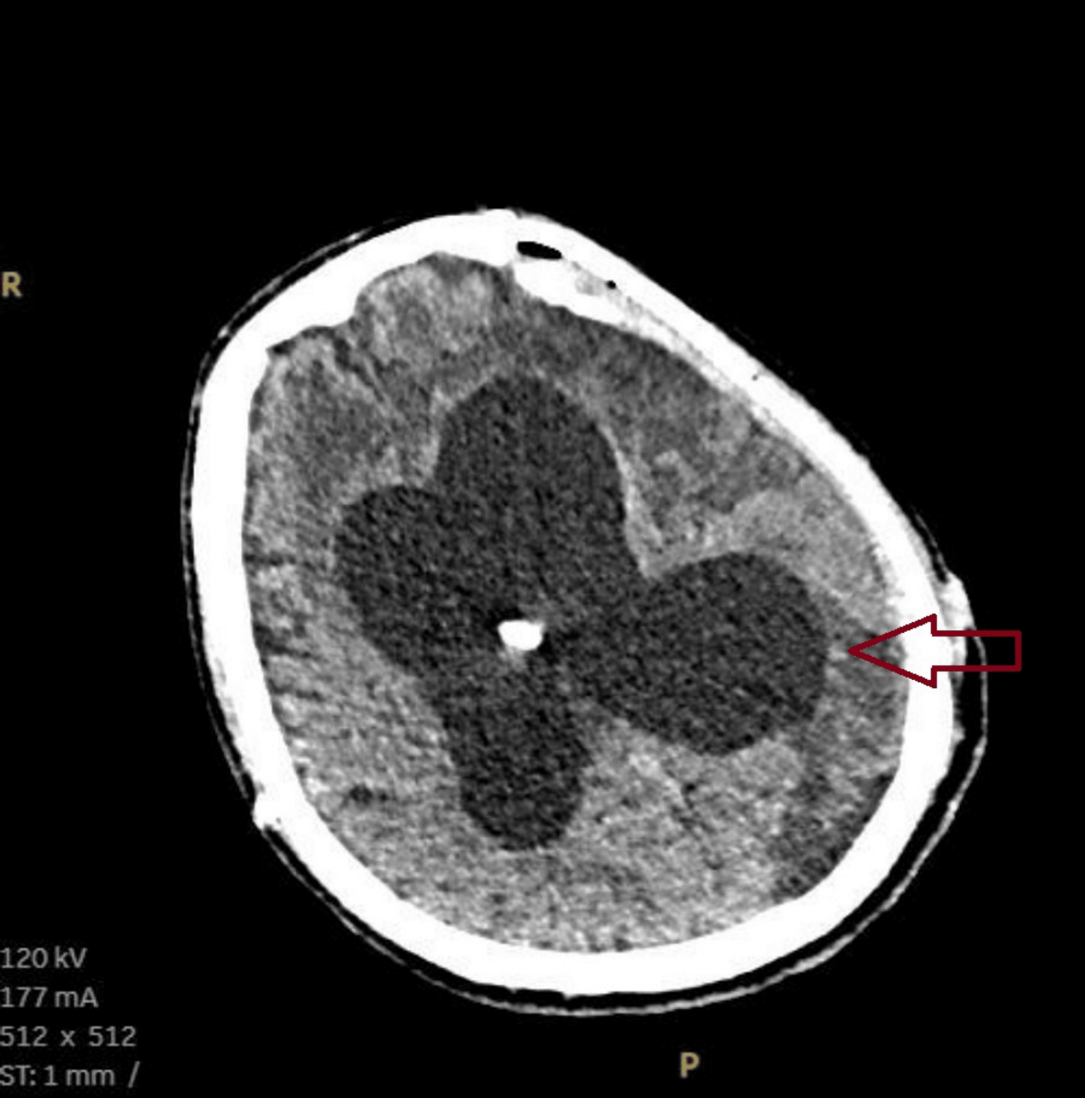
Non-contrast CT brain-enlarged ventricles with VP shunt in situ.

The patient's blood investigation revealed anemia with normal leucocyte counts and, a peripheral smear suggestive of normocytic normochromic anemia with thrombocytosis. The patient likely had anemia of chronic disease in view of his background condition. His renal function test showed elevated creatinine levels (Table 1). The patient’s urine routine showed 15 pus cells. These findings were suggestive of acute kidney injury likely due to a urinary tract infection. His creatinine levels were within normal limits two months back. His blood culture was sterile and urine culture grew pseudomonas. Given acute kidney injury, a contrast computed tomography (CT) brain could not be done and a non-contrast computed tomography of the brain was done which showed enlarged ventricles with VP shunt in situ (Figure 1).

**Table 1:** Laboratory investigation SGOT: Serum glutamic-oxaloacetic transaminase, SGPT: Serum glutamic pyruvic transaminase, ALP: Alkaline phosphatase, GGT: Gamma-glutamyl transferase, TLC: Total leucocyte count, DLC: Differential leucocyte count, RBC: Red blood cell, CSF: Cerebrospinal fluid analysis

Investigation	Values	Reference range
Hemoglobin (g/dl)	9	13-17
Total leucocyte count(cell/cum)	14,000	4-10
Platelet (cell/cumm)	490,000	150-410
Creatinine (mg/dl)	2.1	0.9-1.3
Sodium (mmol/L)	135	136-146
Potassium (mmol/L)	4.6	3.5-5.1
Calcium (mg/dl)	9	8.8-10.2
SGOT (IU/L)	26	15-41
SGPT (IU/L)	28	17-63
ALP (IU/L)	74	32-91
GGT (IU/L)	36	7-50
Albumin (g/dl)	3.3	3.5-5
HbA1c (glycated hemoglobin)	10	<6.5
	CSF Analysis	
Glucose (mg/dl)	62	40-70
Protein (mg/dl)	79	12-60
Lactate (mg/dl)	19.7	0-25
Color	Colorless	
TLC (cells/cumm)	70	<5
DLC	Neutrophil - 15%, Lymphocyte - 85%	
RBC (cell/cumm)	Nil	
Microscopy	Budding yeast and pseudohyphae seen	

**Figure 2:**
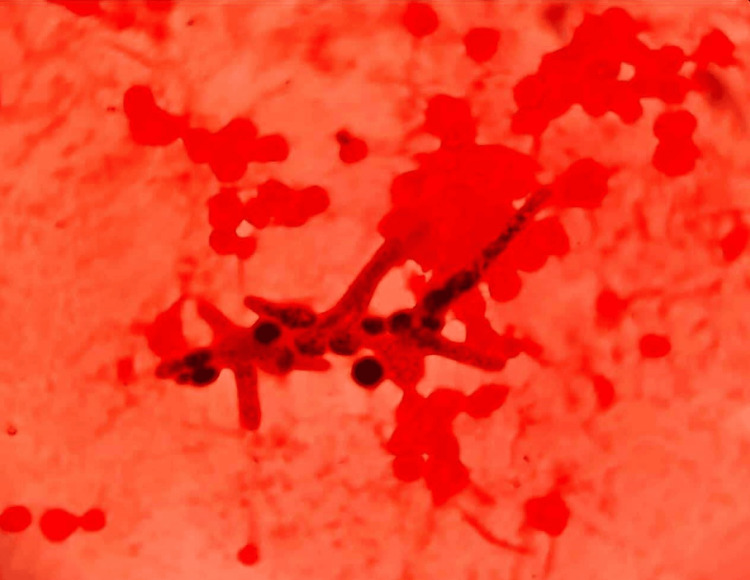
Gram stain of CSF sample- Gram stain of cerebrospinal fluid showing Gram positive budding yeast cells CSF: Cerebrospinal fluid

**Figure 3:**
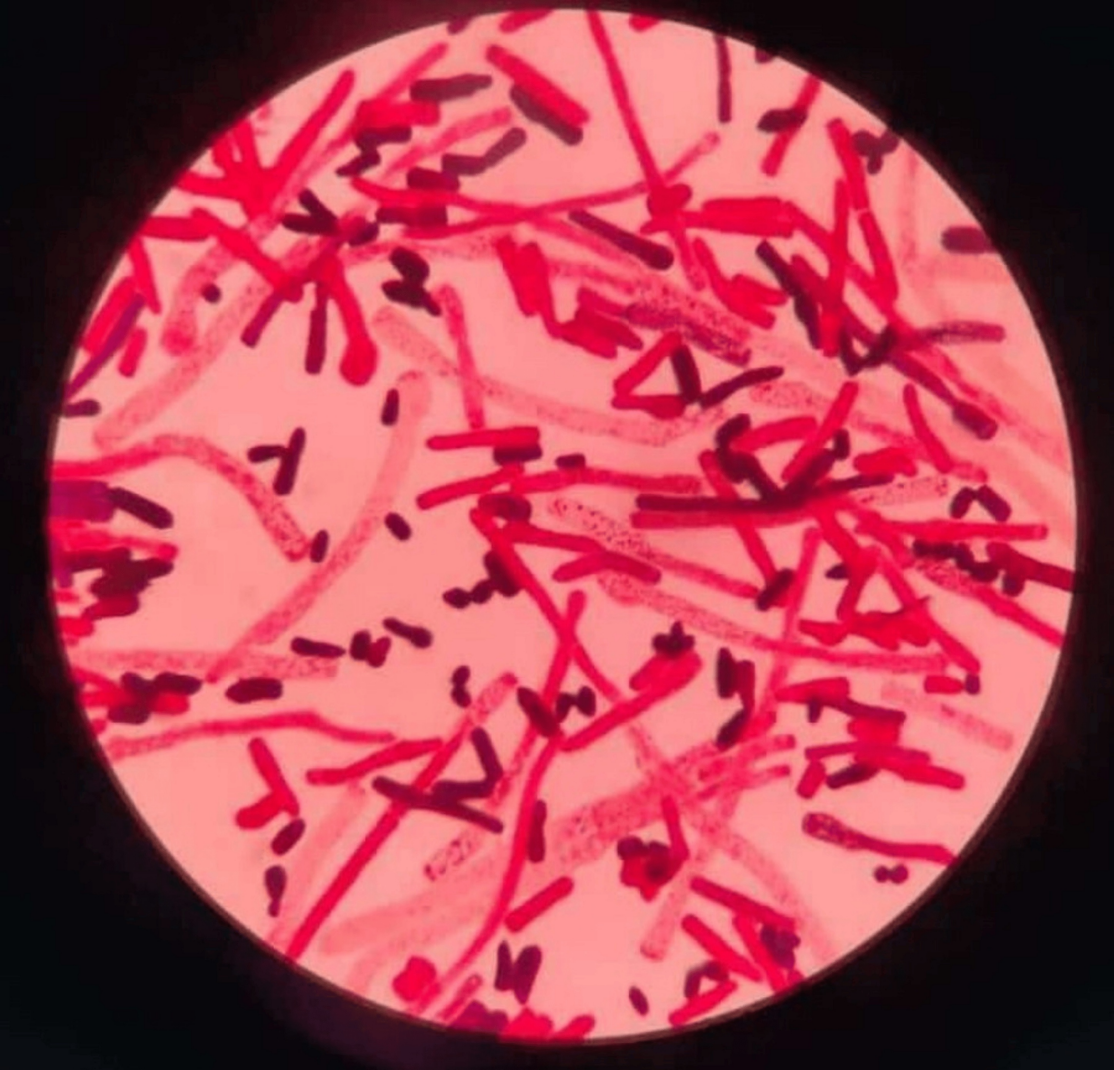
Lactophenol cotton blue of CSF sample- Trichosporon spp. CSF: Cerebrospinal fluid

The patient was suspected to be a case of meningitis with a malfunctioning ventriculoperitoneal shunt. A neurosurgery opinion was taken and the patient was taken up for malfunctioning shunt removal with the insertion of the Ommaya reservoir. His cerebrospinal fluid (CSF) was sent for analysis. The CSF gram stain revealed budding yeast cells and hyphae (Figure 2) with a leucocyte count of 70 cells/cubic mm with 15% neutrophils and 85% lymphocytes, lactophenol cotton blue of CSF sample- Trichosporon spp (Figure 3).

**Figure 4:**
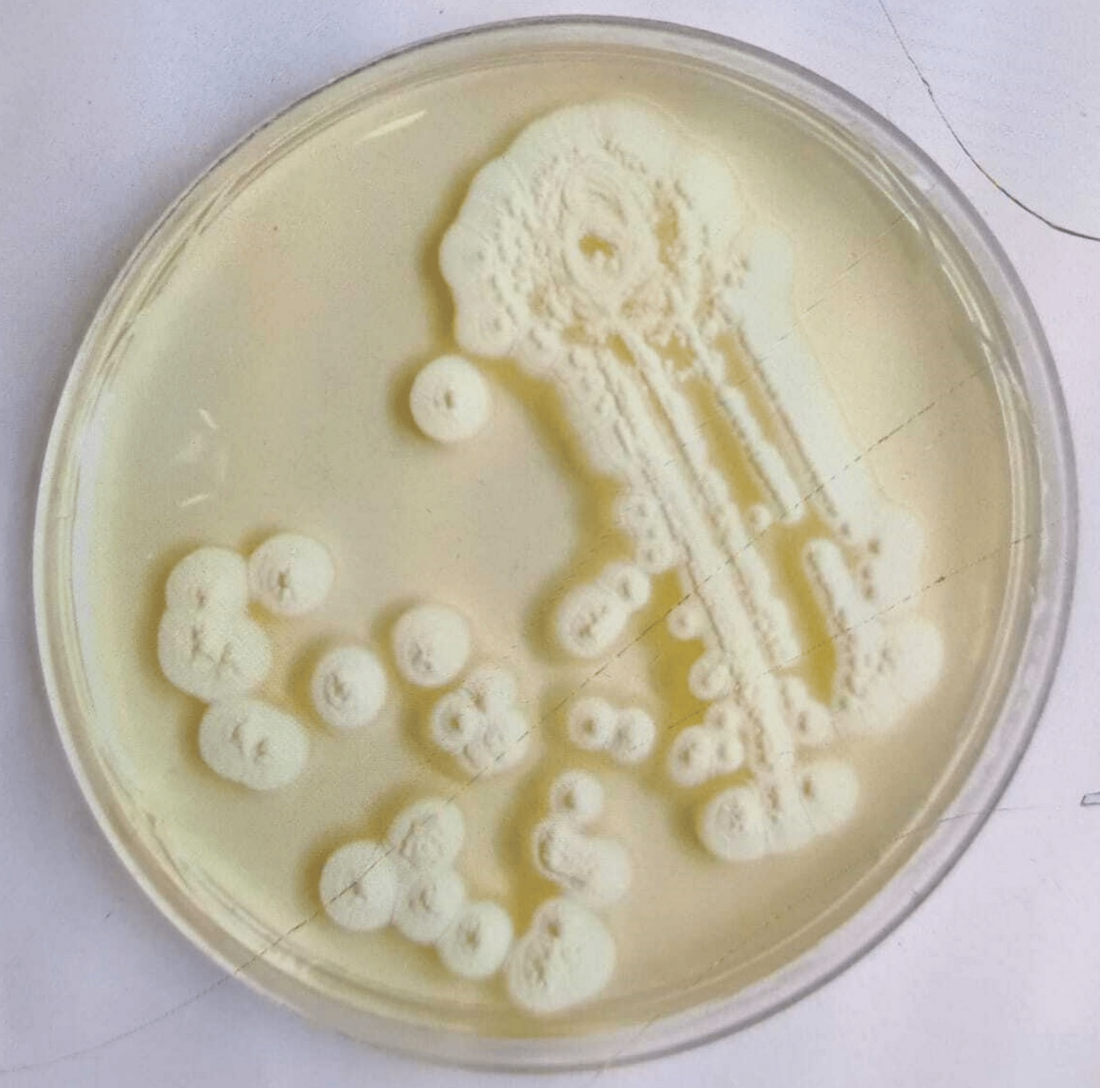
Culture plate of CSF- Growth of Trichosporon spp. on Sabouraud dextrose agar CSF: Cerebrospinal fluid

A MALDITOF-MS (Matrix-assisted laser desorption ionisation - Mass spectrometry) yeast identification revealed Apiotrichum mycotoxinivorans. The CSF biochemistry analysis (Table 1) was exudative suggestive of meningitis. The CSF culture revealed Apiotrichum mycotoxinivorans (Figure 4).

The sensitivity report showed resistance to micafungin, voriconazole, caspofungin, and fluconazole and sensitivity to Amphotericin-B and flucytosine. The CSF showed no acid-fast bacilli on staining and real-time PCR (polymerase chain reaction) for tuberculosis was negative.

Differential diagnosis

The patient was initially suspected to be a case of meningitis with communicating hydrocephalus with a malfunctioning VP shunt. The radiological evidence corroborated the suspicion of the malfunctioning shunt. His CSF analysis revealed findings suggestive of fungal meningitis. The patient culture isolated a rare organism - Apiotrichum mycotoxinivorans which was resistant to most antifungal medications. The organism is found in immunocompromised patients with peritoneal devices. The patient had a background of uncontrolled type 1 diabetes which would contribute to his immunocompromised status. He had a VP shunt in situ for the management of his hydrocephalus.

Treatment

He was initially started on injection piperacillin and tazobactam. His urine culture revealed pseudomonas and was sensitive to piperacillin and tazobactam. This was given for a period of seven days. His blood sugars were managed initially by insulin infusion and he was subsequently started on subcutaneous human insulin injection on a basal bolus regimen. The patient’s fungal meningitis was managed with injectable amphotericin and flucytosine. This is a rare organism and, in this case, it was resistant to most antifungals. The patient was started on Amphotericin and Flucytosine. The VP shunt was removed, an endoscopic third ventriculostomy was done and an Ommaya reservoir was inserted by the neurosurgery team. The patient was stable in the postoperative period. He was given the injectable therapy for six weeks.

Follow up

The patient was followed up after six weeks of treatment. His symptoms had improved. A CSF sample was sent for analysis and was negative for any fungal organism.

## Discussion

This pathogen represents a rare cause of invasive fungal infection in an immunocompromised patient. It is a yeast species that belongs to the kingdom Fungi, the division Basidiomycota, the class Tremellomycetes, the order Trichosporonales, family Trichosporonaceae, genus Apiotrichum, species A. mycotoxinivorans [1]. It is a urease-positive yeast that can grow at 37°C and ferment sugars such as glucose, galactose, maltose, sucrose, and lactose [2]. Apiotrichum mycotoxinivorans can occasionally become a human pathogen, especially in those with compromised immune systems, such as HIV (human immunodeficiency virus infection)/AIDS (acquired immunodeficiency syndrome), cancer, organ transplant, or chemotherapy patients [2]. It can cause various infections, such as respiratory, cutaneous, central nervous system, bloodstream, or disseminated infections. It can also form biofilms on medical devices, such as catheters, prosthetic valves, and implants [2]. We feel this ability of the organism to form biofilm on indwelling catheters or devices is a significant contributor to its pathogenicity. Apiotrichum mycotoxinivorans can express various enzymes, such as proteases, lipases, phospholipases, and esterases, that may help invade and damage the host tissues and cells [2]. It can also produce toxins, such as gliotoxin and trichosporon, that have an immunosuppressive effect [2]. Apiotrichum mycotoxinivorans can evade the immune response by masking its antigens with host proteins, altering its cell wall composition and structure, inducing apoptosis of host cells, and inhibiting the production of pro-inflammatory cytokines [2]. This patient had a background of uncontrolled type 1 diabetes with a history of tuberculosis and a VP shunt in situ for the management of hydrocephalus. All these factors contributed to him contracting this rare fungal infection.

Dabas et al. conducted a study in which a total of 21 Trichosporon spp. isolates from blood over five years (January 2009 to December 2013) were included. The results showed that the most common underlying diseases found were pancreatitis (33.3%) and cancer (33.3%). Trichosporon asahii (80.9%) was the commonest species followed by Trichosporon mycotoxinivorans (14.2%) and Trichosporon faecale (4.7%) [3]. There is no specific serology testing for this fungus. The culture sample from blood, urine, sputum, and cerebrospinal fluid yielded the fungus [2]. In our patient, the CSF culture led to the isolation of this organism.

This is the first time the organism has been isolated in the CSF in India and represents the first case of Fungal meningitis caused by Apiotrichum mycotoxinivorans in India.

At times histopathology samples from the skin can also yield the organism. There are other modalities for molecular identification which are PCR and flow cytometry based but they require further standardization and represent important techniques for further application [2]. The MALDI-TOF-MS method uses a mass spectrometry technique to analyze the protein profiles of the yeast cells. This method can provide rapid and reliable identification [2].

Apiotrichum mycotoxinivorans is resistant to several antifungal agents, and the best antifungal for this infection is not well established. Antifungal susceptibility testing provides invaluable insight into the treatment options. Amphotericin and the azole group have shown activity against Trichosporon spp. in vitro, among these Voriconazole was found to be the most active agent. However, the achievable serum levels of these agents only result in inhibition rather than killing of these fungi [4-8].

In the case of our patient, his antifungal sensitivity report revealed resistance to micafungin, voriconazole, caspofungin, and fluconazole and sensitivity to amphotericin-B and flucytosine. We started the patient on a combination of Amphotericin and Flucytosine. Our review of literature revealed that therapy with Flucytosine has a synergistic effect when combined with amphotericin. We decided to continue combination therapy with these agents for six weeks [9].

We have discussed several interesting case reports. Each of these case reports carries an important message regarding managing infection with this pathogen (Table 2).

**Table 2:** Interesting case reports

S.No.	Author name	Details	Learning message
1.	Almeida JN Jr et al. [10]	A 30-year-old male patient with a history of chronic renal failure came with altered level of consciousness after a peritoneal dialysis session. The patient was diagnosed with sepsis. The peritoneal fluid culture showed Apiotrichum mycotoxinivorans. The patient was treated with amphotericin B deoxycholate, 40 mg/day. His fever persisted, and he developed multiple pulmonary infiltrates. Repeat blood cultures collected on day 12 also showed *Trichosporon* spp. and antifungal therapy was changed to voriconazole on day 14 (300 mg q 12 h on the first day, then 200 mg q 12 h). The attempts to remove the Tenckhoff and haemodialysis catheters were unsuccessful, and the patient’s condition deteriorated due to sepsis by *Chryseobacterium meningosepticum*, and he died on day 29.	This patient is a case of CKD on haemodialysis with catheters in situ. The infection is difficult to treat despite the best antifungal therapy especially in case of indwelling devices.
2	Almeida JN Jr et al. [10]	A 15-year-old boy with a diagnosis of cystic fibrosis. He had a history of multiple pulmonary exacerbations. His latest pulmonary exacerbation was associated with a significant decline in lung function (42 to 28% of predicted forced expiratory volume in the first second - FEV1). His sputum culture became positive for Apiotrichum mycotoxinivorans. His cultures continued to be positive and he was kept on long course therapy with fluconazole (150 mg/day for 21 days). Sputum cultures became negative on days 300, 390, 480. His sputum cultures were positive for *Trichosporon* spp. once again on day 600.	*A. mycotoxinivorans* was first isolated in cases of cystic fibrosis (CF). It may colonise and infect the respiratory tract of CF patients, causing progressive deterioration of their lung function.
3.	Li et al. [11]	A female patient with a background of B-cell acute lymphoblastic leukemia presented as a case of invasive blood and cerebrospinal fluid infection by Apiotrichum mycotoxinivorans. This was the first report of the isolation of Apiotrichum mycotoxinivorans from human cerebrospinal fluid.	This case shows that Apiotrichum mycotoxinivorans can cause invasive cerebrospinal infections.
4	Sadamatsu et al. [4]	A female in her 70s with a background of primary biliary cirrhosis and rheumatoid arthritis was found to have multiple pulmonary nodular shadows on chest radiography. Trichosporon* mycotoxinivorans* and Cryptococcus neoformans were identified in bronchial lavage, and transbronchial lung biopsy specimens. She was treated with fluconazole. Although the pulmonary shadows were under control with treatment, she died 5 months later due to liver failure.	All *Trichosporon* strains can produce measurable levels of GXM antigen, and thus the detection of anticryptococcal cross-reactive antigen may be a useful tool in the early diagnosis of *Trichosporon* infections.

## Conclusions

This is the first time the organism has been isolated in the CSF in India and represents the first case of fungal meningitis caused by Apiotrichum mycotoxinivorans in India. Apiotrichum mycotoxinivorans can occasionally become a human pathogen, especially in those with compromised immune systems, such as HIV/AIDS, cancer, organ transplant, or chemotherapy patients. We feel this ability of the organism to form biofilm on indwelling catheters or devices is a significant contributor to its pathogenicity. The culture samples from blood, urine, sputum, and cerebrospinal fluid are the best diagnostic modality. MALDITOF-MS yeast identification is a useful tool for diagnosis. Amphotericin and the azole group have shown activity against Trichosporon spp. in vitro. Among these voriconazole was found to be the most active agent. A high death rate is associated with Apiotrichum mycotoxinivorans infection which ranges from 25% to 100%, depending on the type and severity of the infection and the patient’s underlying conditions. Further studies are required to establish a standard line of management for this uncommon organism.
